# The Sequence Characteristics and Binding Properties of the Odorant-Binding Protein SvelOBP1 from *Sympiezomias velatus* (Coleoptera: Curculionidae) to Jujube Volatiles

**DOI:** 10.3390/life14020192

**Published:** 2024-01-29

**Authors:** Yingyan Zhai, Feng Zhang, Tianqi Tian, Yiwei Yang, Yang Li, Bowen Ren, Bo Hong

**Affiliations:** 1 Shaanxi Key Laboratory of Qinling Ecological Security, Bio-Agriculture Institute of Shaanxi, Shaanxi Academy of Sciences, Xi’an 710043, China; zhaiyy@xab.ac.cn (Y.Z.); zhangfeng73@xab.ac.cn (F.Z.); tiantianqi@xab.ac.cn (T.T.); yangyw@xab.ac.cn (Y.Y.); 2 Chang’an University Journal Center, Chang’an University, Xi’an 710064, China; li.yang@chd.edu.cn; 3 Institute of Forest Protection, Shaanxi Academy of Forestry, Xi’an 710016, China; jones193@126.com

**Keywords:** odorant reception, odorant-binding proteins, *Sympiezomias velatus*, host plant volatile, binding affinity, molecular docking

## Abstract

*Sympiezomias velatus* (Chevrolat) (Coleoptera: Curculionidae) has caused serious damage on jujube trees (*Ziziphus jujuba* Mill) in northern China. Semiochemicals emerging from the host are essential in the process of insects identifying and localizing the host. The highly expressed odorant-binding protein 1 of *S. velatus* (SvelOBP1) was assumed to play a possible role in the recognition of host volatiles. In this study, *SvelOBP*1 was cloned based on the antennal transcriptome of *S. velatus*. The recombinant SvelOBP1 protein was expressed in *Escherichia coli* and purified by Ni-NTA resin. The predicted protein SvelOBP1 belonged to a classic OBP subfamily. The expression patterns revealed that *SvelOBP*1 was mainly expressed in the antennae of both males and females, whereas the expression of SvelOBP1 in other body parts could be neglected. The fluorescence binding assay indicated that SvelOBP1 displayed very strong binding affinities to dibutyl benzene-1,2-dicarboxylate and (*Z*)-hex-3-en-1-ol (K_i_ = 6.66 ± 0.03 and 7.98 ± 0.06 μM). The molecular docking results showed that residues Trp114, Phe115 and Asp110 may be involved in binding to both dibutyl benzene-1,2-dicarboxylate and (*Z*)-hex-3-en-1-ol and may have a great impact on odorant recognition of *S. velatus*. Our results provide evidence that SvelOBP1 might participate in the olfactory molecular perception of *S. velatus* and would promote the development of pest attractants for *S. velatus* control.

## 1. Introduction

The great gray weevil, *Sympiezomias velatus* (Chevrolat) (Coleoptera: Curculionidae), is widely distributed in northern China. *S. velatus* is an omnivorous agricultural pest which feeds on many trees and crops, such as jujube, apple, elm, peanut, maize, cotton and so on [1,2]. *S. velatus* has caused serious damage on jujube trees (*Ziziphus jujuba* Mill) at the junction of Shaanxi and Shanxi Provinces along the Yellow River [1,3]. To control *S.velatus*, chemical pesticides are predominantly used [4]. The long-term use of pesticides will cause insect resistance and environmental pollution.

The insect olfactory system is indispensable for various important behaviors, such as foraging, mating and spawning [5,6]. The olfactory perception process is a highly complex chain reaction involving interactions among odorant molecules and various olfactory proteins, such as odorant-binding proteins (OBPs), olfactory receptors (ORs), ionotropic receptors (IRs) and sensory neuron membrane proteins (SNMPs) [7,8,9,10]. OBPs are responsible for odorant reception in the early stages of chemosensory systems. They are the liaison between the external environment and ORs [11]. Once the hydrophobic odorant molecules move through the pore tubules and across the sensillar lymph, OBPs recognize, bind to and transport odorant molecules to ORs, which localize on the membrane of olfactory receptor neurons (ORNs). Afterwards, the signal transduction to the central nervous system is activated, and various behaviors will finally act based on different odors [12,13,14].

OBPs have attracted a research upsurge because of their large family and complex functions. The sizes of OBPs are usually in the range of around 100–200 amino acids and they have a molecular mass of 12–20 kDa [15]. Based on the number of conserved cysteine (Cys) residues, insect OBPs can be divided into five subfamilies: classic OBPs (six conserved cysteines), plus-C OBPs (eight conserved cysteines), minus-C OBPs (four conserved cysteines), dimer OBPs (twelve conserved cysteines) and atypical OBPs (nine to ten conserved cysteines) [16,17]. Since the first OBP was found in *Antheraea polyphemus* [18], more OBPs have been identified based on the transcriptome sequencing technique in recent years [19,20,21,22,23,24,25,26]. Collectively, these studies illustrated that OBPs, which were highly expressed in antennae as well as other body parts (e.g., legs, wings and larval maxilla), showed specific binding affinities with different host plant volatiles, and this phenomenon indicated that OBPs have the function of orientation and host selection at the molecular level. It is well known that a protein function is specified by its structure. OBPs have conservative Cys residues and compact α-helical domains. The accumulated evidence indicates that hydrogen bonds formed by amino acid residues with the functional groups of ligands are the main binding force, but there are also hydrophobic interactions or Van Der Waals interactions involved in the molecular binding process [27,28,29].

OBPs are potential molecular targets for finding novel lures for pest control [30]. Although SvelOBP15 was identified and its binding affinities have been quantified [31], our knowledge of OBPs in *S. velatus* is still insufficient and more investigations are required. The fragments per kilobase of transcript per million mapped read (FPKM) values of *SvelOBP*1 were highest based on the transcriptome of *S. velatus* antennae (the sequence of *SvelOBP1* in this study is consistent with *SvelOBP37* in Li’s study) [31]. Based on this conclusion, we hypothesized that *SvelOBP*1 is an antennae-enriched OBP gene which is related to host plant recognition. In this study, the *SvelOBP*1 gene was selected and identified, and the expression profile was also determined in different body parts of both sexes. After purifying the recombined protein, the binding assay was performed on SvelOBP1 with 43 jujube volatiles. Three-dimensional structural modeling and molecular docking were also used to identify the key amino acid residues that contributed to the binding interactions with the SvelOBP1 binding sites. Our results will further enrich the gene inventory of *S. velatus* and provide potential targets for new pest control strategies.

## 2. Materials and Methods

### 2.1. Insect Culture, Body Part Collection and RNA Isolation

The tested pupae of *S. velatus* were collected from Yongji County, Shanxi Province, China (110.60° E, 34.95° N), in April 2021, and incubated in an artificial climate chamber at 25 ± 1 °C, 60 ± 5% RH, and 16 h light: 8 h dark cycle. The emerged adults were separated into males and females and fed with fresh jujube buds. Different body parts were separated from 5-day-old adults of *S. velatus*, including antennae, heads without antennae, thoraxes, abdomens, legs and wings. Fresh body parts were immediately transferred to 1.5 mL centrifuge tubes and stored at −80 °C.

Total RNA was extracted from different body parts of both sexes with RNAiso Plus reagent (TaKaRa, Dalian, China). The integrity and quantity of RNA samples were tested by using gel electrophoresis and a spectrophotometer (Nanodrop-500, Allsheng, Hangzhou, China), respectively. First-strand complementary DNA (cDNA) was synthesized from 1.0 μg of total RNA using PrimeScript^TM^ 1st Strand cDNA Synthesis Kit (TaKaRa) following the recommended protocols from the kit, and cDNA samples were stored at −20 °C.

### 2.2. cDNA Synthesis and Gene Cloning

Open reading frame (ORF) of the putative *SvelOBP*1 was determined by ORF Finder https://www.ncbi.nlm.nih.gov/orffinder/ (accessed on 3 March 2023). Degenerate primers were designed to clone the *SvelOBP*1 gene based on the homologous sequence from *Pachyrhinus yasumatsui* [32] and *Sitophilus zeamais* [33] (Table 1). A polymerase chain reaction (PCR) was carried out by using 2xTaq MasterMix (Cwbio biotech, Beijing, China) with a template of male antennae cDNA. The reaction environmental conditions were as follows: 94 °C for 3 min, 35 cycles of 94 °C for 30 s, 55 °C for 30 s, 72 °C for 30 s, and with a final extension for 10 min. After being separated by electrophoresis and purified with the gel extraction kit (Tiangen, Beijing, China), the PCR products were ligated into pMD^TM^ 19-T vector and transferred into *E. coli* DH5*α*cells (Tiangen). Cells were screened by means of blue–white selection [34,35]. Positive overnight-grown clones were incubated with ampicillin (100 mg/mL) at 220 rpm/min, 37 °C. Three positive clone plasmids were extracted by TIANprep Mini Plasmid Kit (Tiangen, Beijing, China) and were subjected to sequencing by Sangon Biotech Company (Shanghai, China).

### 2.3. Sequence Alignment and Phylogenetic Analyses

The physical and chemical parameters of SvelOBP1 were calculated using ExPASy tool https://web.expasy.org/protparam/ (accessed on 15 March 2023). The signal peptides at the N-terminus were predicted by SignalP-6.0 server with default parameters https://services.healthtech.dtu.dk/services/SignalP-6.0/ (accessed on 15 March 2023). The secondary structure of SvelOBP1 protein was predicted by using SOPMA prediction method https://npsa-prabi.ibcp.fr/cgi-bin/npsa_automat.pl?page=npsa%20_sopma.html (accessed on 20 March 2023).

The sequence similarity of SvelOBP1 protein was determined by BLASTp https://blast.ncbi.nlm.nih.gov/Blast.cgi (accessed on 1 April 2023). Conserved cysteines were searched for by WebLogo 3 Online https://weblogo.threeplusone.com/create.cgi (accessed on 30 December 2023) based on the amino acid sequence alignment of SvelOBP1 and other homologous OBP sequences. A total of 39 full-length amino acid sequences of 16 insects from coleopteran were aligned by MEGA-X. The phylogenetic analysis was constructed based on the neighbor-joining method in order to assess the systematical evolution among different species. Figtree 1.4.4 http://tree.bio.ed.ac.uk/software/figtree/ (accessed on 10 May 2023) was used to edit the phylogenetic tree.

### 2.4. RT-qPCR Detection for Body Part Expression of SvelOBP1

The expression profile of *SvelOBP*1 in different body parts was tested by quantitative real-time polymerase chain reaction (qRT-PCR) with a StepOnePlus Real-Time PCR System (ABI, Carlsbad, CA, USA). The *β-actin* gene (GenBank No. OM417063) and *elongation factor 1α* gene *(EF-1α)* (GenBank No. MH717233) of *S. velatus* were used as reference genes. Specific primers were designed by Primer3 web (version 4.1.0) https://primer3.ut.ee (accessed on 8 April 2023) and are shown in Table 1. Each PCR volume (20 μL) contained 100 ng of cDNA template from different body parts of both sexes. The reaction environmental conditions were as follows: 95 °C for 30 s, 40 cycles of 95 °C for 5 s, 60 °C for 30 s and 72 °C for 30 s. All experiments were performed with three biological replicates and three technical repeats. The relative expression levels of *SvelOBP*1 in different adult body parts were analyzed using the 2^−△△Ct^ method [36]. The expression level from the female abdomen was used as the standard to calculate the expression level of other body parts. A one-way ANOVA with Tukey’s post hoc test was used for the calculation of significant differences between different body parts of SvelOBP1. Student’s *t*-test was used to compare the expression between both sexes in the same body part of SvelOBP1. The data were analyzed by SPSS Statistics 22.0.

### 2.5. Recombinant Plasmid Construction for E. coli Expression

Specific primers were designed with restriction enzyme sites *BamH*I and *EcoR*I by Primer Premier 5.0 http://www.premierbiosoft.com/primerdesign/ (accessed on 12 April 2023) (Table 1). The cDNA-encoding mature SvelOBP1 without signal peptides was cloned by PCR amplification. The PCR products were isolated and ligated into pMD^TM^ 19-T cloning vector, and then transferred into *E. coli* DH5*α* cells. The positive clone plasmids were extracted by TIANprep Mini Plasmid Kit. The plasmids and expression vector pET32a (+) (TaKaRa) were separated digested with restriction enzymes *BamH*I and *EcoR*I (TaKaRa) for 1 h at 37 °C. Digestion reactions of 50 μL were performed as follows: 12.5 μL plasmid of target gene or vector pET32a (+), 5 μL FastDigest Green buffer, 5 μL *BamH*I, 5 μL *EcoR*I and 22.5 μL ddH_2_O, and then, SvelOBP1 constructs were ligated into digested pET32a (+). The ligation products were transformed into competent DH5*α* cells and the target sequences were verified. The positive recombinant plasmids were transferred into *E. coli* BL21 (DE3) competent cells (TaKaRa) for target protein expression.

### 2.6. Expression and Purification of Recombinant SvelOBP1 Protein

The positive single clone was inoculated in LB liquid medium with ampicillin (100 mg/mL) and grown overnight at 37 °C. The culture was diluted with fresh LB liquid medium at a 1:100 ratio and incubated until OD_600_ reached 0.6–0.8. A total of 0.5 mM Isopropyl-β-D-1-thiogalactopyranoside (IPTG) was added into the culture to induce the expression of the recombinant protein and incubated at 20 °C for 10 h. The cells (500 mL) were collected by centrifugation at 8000 rpm, 4 °C, for 10 min. The cell pellets were suspended in 20 mM Tris-HCl and 50 mg/mL Lysozyme at pH7.4 for 30 min. The suspension was sonicated on ice for 10 min and centrifuged at 12,000 rpm, 4 °C, for 30 min. The supernatant and pellet were collected and tested by 15% SDS-PAGE. The target protein in the supernatant was further purified and separated with a pre-equilibrated column with Ni-NTA His-Bind Resin (7Sea, Shanghai, China). The protein sample was loaded into the column, and the column was washed by 10 mL equilibrium buffer (20 mM Tris-HCl, pH7.4, 250 mM NaCl and 1 mM imidazole), followed by 10 mL wash buffer (20 mM Tris-HCl, pH7.4, 250 mM NaCl and 20 mM imidazole) to remove the non-target protein. After that, SvelOBP1 was eluted by 10 mL elution buffer (20 mM Tris-HCl, pH7.4, and 250 mM NaCl) with concentrations of 50, 100, 200 and 250 mM imidazole, respectively. Each eluate was collected by centrifuge tubes and assessed by SDS-PAGE. The 200 mM elution fractions containing the highly purified target protein were desalted through dialysis in 20 mM Tris-HCl buffer (pH7.4) for 24 h at 4 °C and then stored at −20 °C. The concentration of the protein solution was quantified following by the protocols of BCA protein assay kit (Cwbio Biotech, Beijing, China). The concentration of the pET32a (+)/SvelOBP1 protein solution after purification and elution was 1.75 mg/mL. His-tag was removed to avoid the confounding effects by using recombinant enterokinase (rEK) (Yeasen, Shanghai, China). The mixture of recombinant protein and rEK was incubated at 25 °C for 16–24 h to ensure complete digestion. The concentration of the SvelOBP1 protein solution after tag removal was 0.33 mg/mL.

### 2.7. Competitive Fluorescence Binding Assay

The fluorescent probe 1-N-phenyl-naphthylamine (1-NPN) [13,25,37,38] was used to evaluate the binding affinity of SvelOBP1 protein to different ligands. Forty-three host plant volatiles from leaves of jujube trees were selected as putative ligands (Table 2) [39,40]. Fluorescence-based ligand binding assays were performed on an F-2700 fluorescence spectrophotometer (Hitachi, Tokyo, Japan). The excitation wavelength was 337 nm, and the emission spectra were in the range from 370 to 550 nm. First, all tested ligands and 1-NPN were dissolved in chromatographic methanol and diluted to 1 mM as the stock solution. Second, the recombinant protein was diluted to 2 μM with 20 mM Tris-HCl (pH7.4), and 2 mL of the protein solution was placed in a 1 cm light path quartz cuvette.

To determine the binding affinity of 1-NPN for SvelOBP1 protein, the protein solution was titrated with aliquots of 1 mM 1-NPN solution to obtain final concentrations of 0–16 μM, and the standard curve was generated based on the intensity values of three replicates corresponding to the maximum fluorescence emission. To measure the protein binding affinities, each ligand solution (1 mM) was added as aliquots into the 2 μM SvelOBP1/1-NPN mixture to obtain final concentrations ranging from 0 to 16 μM to replace 1-NPN, and then, the competitive binding curves were plotted based on the fluorescence intensity values of the three replicates. The dissociation constants K_1-NPN_ of 1-NPN to SvelOBP1 were calculated by GraphPad Prism 8.0 software with non-linear regression for the one site-specific binding method [41]. The inhibitory constant (K_i_) of each ligand binding to SvelOBP1 was calculated with Equation (1) [22]. IC_50_ is the ligand concentration replacing 50% of the initial fluorescence intensity of the SvelOBP1/1-NPN mixture. [1-NPN] represents the free concentration of 1-NPN. The binding affinity of ligands to SvelOBP1 is considered very strong (K_i_ ≤ 5 μM), strong (5 μM < K_i_ ≤ 10 μM), moderate (10 μM < K_i_ ≤ 16 μM) and weak (K_i_ > 16 μM).

(1) $$K_{i}=\left[{IC}_{50}\right]/(1+[1-NPN]/K_{1-NPN})$$

### 2.8. Structure Modeling and Molecular Docking

The structural templates of SvelOBP1 were searched for using SWISS-MODEL Repository https://swissmodel.expasy.org/interactive (accessed on 5 June 2023). *Anopheles gambiae* OBP1 (AgamOBP1; PDB ID: 2ERB) was chosen to be the template based on the similarity and coverage with the SvelOBP1 amino acid sequence without a signal peptide. The homology modeling was performed using Modeller 10.4 [32,42]. Verify3D and PROCHECK software https://saves.mbi.ucla.edu/ (accessed on 11 June 2023) were applied to assess the reliable three-dimensional (3D) structure of SvelOBP1. The molecular conformations of ligands were predicted and optimized by ChemBioDraw 12.0 [43]. The molecular docking between SvelOBP1 and ligands was performed using Autodock v.4.2.6. The visualization of 2D and 3D structures was executed by using Discovery Studio 2019 (BIOVIA) and PyMOL v.2.6.0 (Schrödinger).

## 3. Results

### 3.1. Characterization of SvelOBP1 cDNA

The full-length cDNA of *SvelOBP*1 (GenBank No. OQ740559) was cloned by RT-PCR. The ORF of *SvelOBP*1 was 408 bp encoding 135 amino acids, with a predicted MW of 15.4 kDa and a theoretical pI of 4.73. SvelOBP1 possessed a predicted signal peptide with 18 residues at the N-terminus (Figure S1).

**Figure 1:**
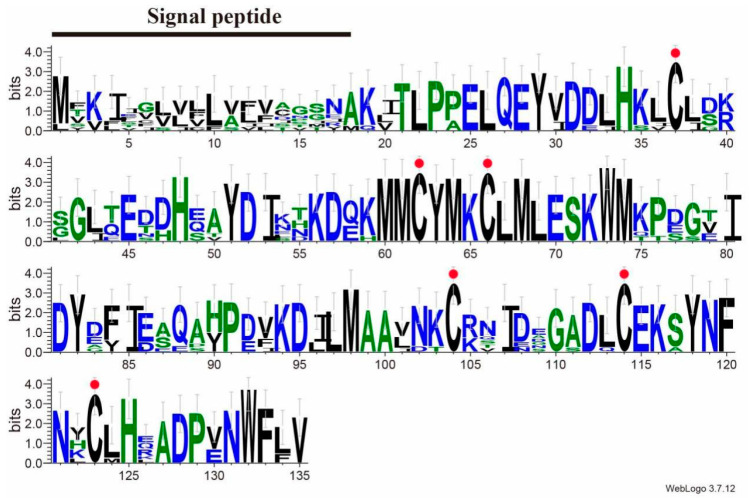
OBP1 sequence alignment of *S. velatus* and other coleopteran species. The total height of the stack represents the conservation of sequence at the site. The height of each letter shows the relative frequency of each amino acid at the site. Conserved cysteine residues are highlighted with red dots. OBPs from different species and their GenBank accession numbers are as follows: PyasOBP2 (UUK33715.1) from *Pachyrhinus yasumatsui*, AgraPBP6 (XP_050315177.1) and AgraGOBP83a (XP_050315178.1) from *Anthonomus grandis grandis*, PtsuOBP25 (UWL63315.1) from *Pagiophloeus tsushimanus*, RferGOBP83a (KAF7281467.1) from *Rhynchophorus ferrugineus*, DarmOBP2 (AIY61045.1) from *Dendroctonus armandi,* SzeaOBP8 (QCT83262.1) from *Sitophilus zeamais,* and SoryGOBP83a (XP_030747957.1) from *Sitophilus oryzae*.

**Figure 2:**
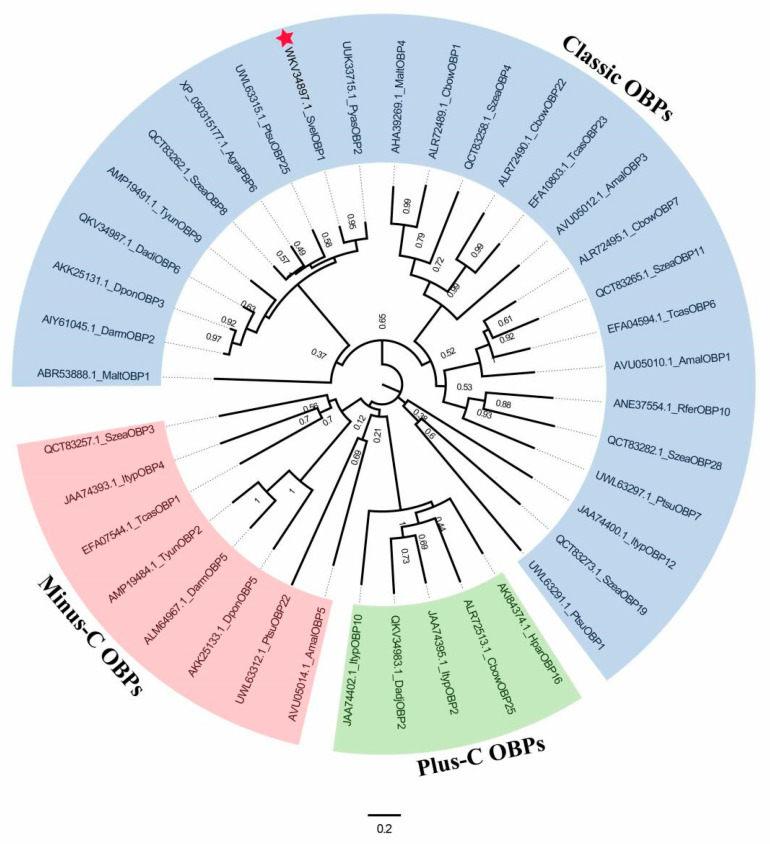
The phylogenetic tree of 39 odorant-binding proteins (OBPs). SvelOBP1 is indicated by a red star. Gene names of 39 OBPs from coleopteran insects are as follows: *Anthonomus grandis grandis* (AgraPBP6), *Agrilus mali* (AmalOBP1, AmalOBP3 and AmalOBP5), *Colaphellus bowringi* (CbowOBP1, CbowOBP7, CbowOBP22 and CbowOBP25), *Dendroctonus armandi* (DarmOBP2 and DarmOBP5), *Dendroctonus adjunctus* (DadjOBP2 and DadjOBP6), *Dendroctonus ponderosae* (DponOBP3 and DponOBP5), *Holotrichia parallela* (HparOBP16), *Ips typographus* (ItypOBP2, ItypOBP4, ItypOBP10 and ItypOBP12), *Monochamus alternatus* (MaltOBP1 and MaltOBP4), *Pagiophloeus tsushimanus* (PtsuOBP1, PtsuOBP7, PtsuOBP22 and PtsuOBP25), *Pachyrhinus yasumatsui* (PyasOBP2), *Rhynchophorus ferrugineus* (RferOBP10), *Sympiezomias velatus* (SvelOBP1), *Sitophilus zeamais* (SzeaOBP3, SzeaOBP4, SzeaOBP8, SzeaOBP11, SzeaOBP19 and SzeaOBP28), *Tribolium castaneum* (TcasOBP1, TcasOBP6 and TcasOBP23), and *Tomicus yunnanensis* (TyunOBP2 and TyunOBP9).

BLAST analysis showed that SvelOBP1 shared relatively higher amino acid sequence identity of 80.74% with PyasOBP2 from *Pachyrhinus yasumatsui* followed by 76.27% with SzeaOBP8 from *Sitophilus zeamais*, then 73.33% with PtsuOBP25 from *Pagiophloeus tsushimanus* and 70.23% with DarmOBP2 from *Dendroctonus armandi*. Based on the results of the sequence alignments of SvelOBP1 with eight homologous OBPs (Figure 1), SvelOBP1 exhibited typical characteristics of the classic OBP subfamily, including approximately 150 amino acids with six conserved Cys residues [17]. Phylogenetic analyses suggested that 38 OBPs from other coleopteran insects were divided clearly into three subfamilies: classic OBPs, minus-C OBPs and plus-C OBPs. SvelOBP1 was clustered into the classic OBP family (Figure 2) and SvelOBP1 was closest to PyasOBP2, which was consistent with the BLAST analysis.

### 3.2. Body Part Expression Profiles of SvelOBP1

**Figure 3:**
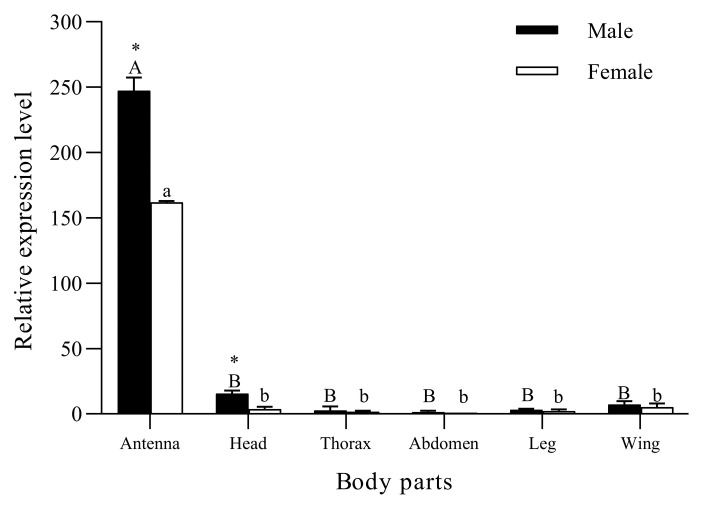
The expression profiling of *SvelOBP*1 in different body parts of *S. velatus* males and females. The expression level of female abdomen was used as the standard to calculate the expression level of other body parts. Different lowercase and capital letters above the bars indicate the significant difference between different body parts of male and female adults. Asterisks above the letters denote significant difference in the expression levels of *SvelOBP*1 in the same body parts between males with females.

The RT-qPCR results (Figure 3) show that *SvelOBP*1 was significantly expressed in the antennae compared with other body parts in both male and female adults of *S. velatus* (♂: *F*_5, 12_ = 1080.46, *p* < 0.001; ♀: *F*_5, 12_ = 3103.04, *p* < 0.001). *SvelOBP1* was negligibly expressed in the thoraxes, abdomens, legs and wings in both sexes. The expression levels of *SvelOBP*1 in the antennae (t = 114.74, *p* < 0.01) and heads (t = 7.26, *p* < 0.01) were significantly higher in males than those in females. There were no significant differences found in the thoraxes, abdomens, legs and wings between males and females.

### 3.3. Expression and Purification of SvelOBP1

**Figure 4:**
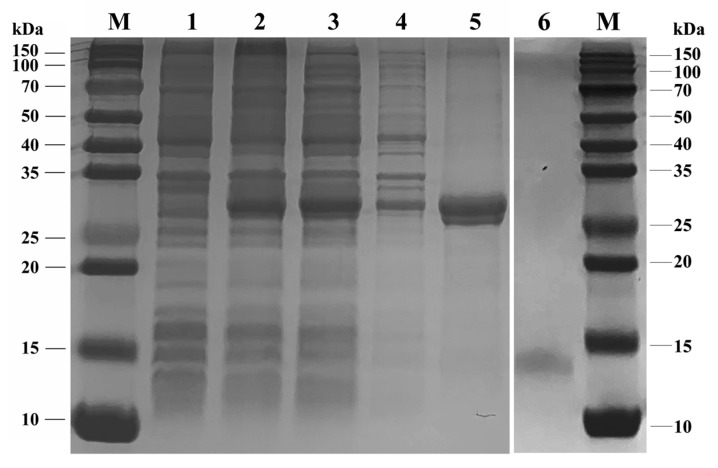
SDS-PAGE analysis of recombinant SvelOBP1 protein. M: protein molecular weight marker. 1: The pET32a (+)/SvelOBP1 protein without induction. 2: The pET32a (+)/SvelOBP1 protein with IPTG induction. 3: The supernatant of pET32a (+)/SvelOBP1 protein. 4: The pellet of pET32a (+)/SvelOBP1 protein. 5: The purified pET32a (+)/SvelOBP1 protein. 6: The SvelOBP1 protein after removing the His-tag.

The analyses of the SDS-PAGE assay (Figure 4) indicated that the recombinant SvelOBP1 was successfully expressed in the prokaryotic expression system. It was shown that the recombinant SvelOBP1 protein was mostly found in the supernatant. The size of the purified and enriched recombinant SvelOBP1 protein was about 30 kDa. The concentration and the molecular weight of the target SvelOBP1 protein was 0.33 mg/mL and 14 kDa after the successful removal of His-tag through rEK digestion.

### 3.4. Fluorescence Competitive Binding Analyses of SvelOBP1

**Figure 5:**
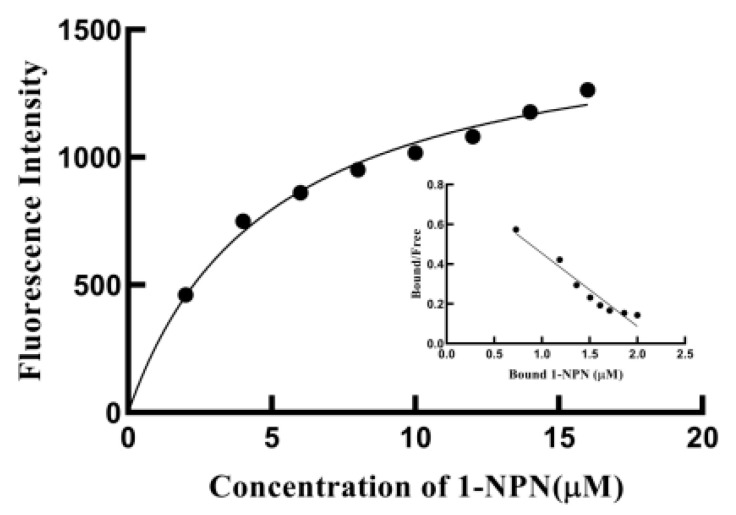
Binding curves for 1-NPN and the linear Scatchard plot.

**Figure 6:**
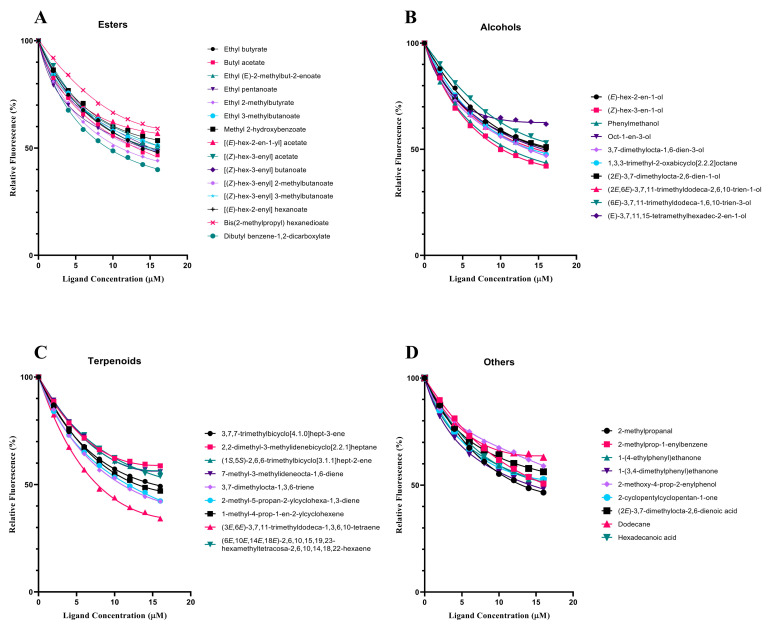
The binding assays of 43 volatiles for recombinant SvelOBP1. The K_i_ values of SvelOBP1 for different tested compounds are listed in Table 2. Aldehydes: 2-methylpropanal; alkenes: 2-methylprop-1-enylbenzene; alkanes: dodecane; ketones: 1-(4-ethylphenyl)ethanone, 1-(3,4-dimethylphenyl)ethanone, and 2-cyclopentylcyclopentan-1-one; phenols: 2-methoxy-4-prop-2-enylphenol; acids: (2*E*)-3,7-dimethylocta-2,6-dienoic acid and hexadecanoic acid.

In order to test the function of SvelOBP1 in the perception of jujube leaf volatiles, the binding activities of a total of 43 putative ligands to the recombinant protein SvelOBP1 were tested. The binding constant of SvelOBP1 to 1-NPN was analyzed, and the Scatchard plot was created (Figure 5). The dissociation constant (K_d_) was 4.92 ± 0.04 μM when SvelOBP1 bound with 1-NPN, suggesting that 1-NPN was a suitable fluorescent reporter in the binding analyses. The competitive binding results are exhibited in Table 2 and Figure 6. SvelOBP1 was found to be able to bind to 24 compounds (K_i_ values < 16 μM) [41,44] among the 43 tested odorants. SvelOBP1 exhibited high binding affinities to ten esters (K_i_ values from 6.66 ± 0.03 to 12.90 ± 0.75 μM) and seven alcohols (K_i_ values from 7.98 ± 0.08 to 12.83 ± 0.12 μM) (Figure 6A,B), and it could bind as well to five terpenoids (K_i_ values from 8.73 ± 0.09 to 12.10 ± 0.06 μM) (Figure 6C). According to the binding test results, we believe that SvelOBP1 displayed a broad binding profile, being able to bind to ligands with different structural characteristics. Additionally, SvelOBP1 had moderate binding affinities for the ketone 1-(4-ethylphenyl)ethanone (10.91 ± 0.13 μM) and the acid hexadecanoic acid (11.71 ± 0.27 μM) in the category of “others” (Figure 6D). Among all tested compounds, the ester dibutyl benzene-1,2-dicarboxylate and the alcohol (*Z*)-hex-3-en-1-ol displayed the strongest binding affinity for SvelOBP1 (K_i_ = 6.66 ± 0.03 and 7.98 ± 0.08 μM).

### 3.5. Three-Dimensional Modeling and Molecular Docking of SvelOBP1

**Figure 7:**
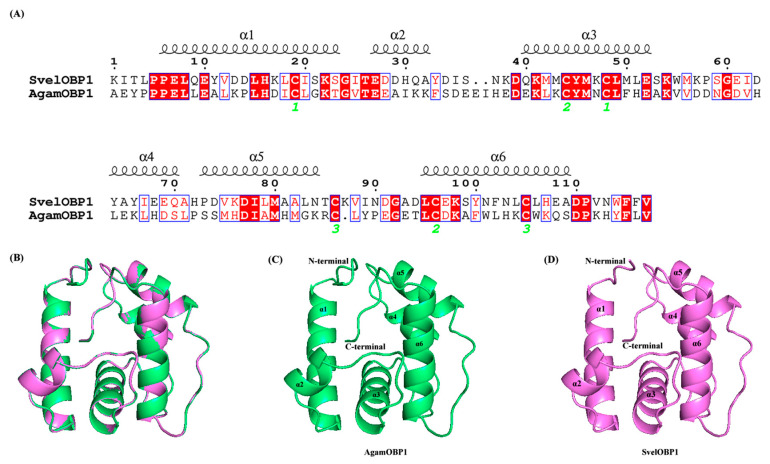
Three-dimensional structure modeling. (**A**) The amino acid sequence alignment of template protein AgamOBP1 (ID: 2ERB) and target protein SvelOBP1. The cysteines which formed disulfide bridges are labeled by green digits below the sequence. (**B**) The superimposed structure of SvelOBP1 and AgamOBP1. (**C**) The three-dimensional structure of template protein AgamOBP1. (**D**) The predicted three-dimensional structure of target protein SvelOBP1.

According to SOPMA prediction results, the secondary structure of SvelOBP1 contained 55.56% *α*-helices and 31.11% random coil, in addition to 8.15% extended strand and 5.19% *β*-turn. SvelOBP1 has manifested a high sequence similarity (76.07%) and coverage (96.00%) with AgamOBP1 (PDB ID: 2ERB), as shown in Figure 7A. The 3D structure of SvelOBP1 was constructed by using 2ERB as the homology modeling template, and the predicted model was assessed by Verify 3D and PROCHECK. The analyses of Verify 3D showed that 82.05% of SvelOBP1 residues scored above 0.2. A total of 96.3% of residues were within the favorable region according to the Ramachandran plot analysis (Figures S2 and S3). Therefore, the 3D modeling result of SvelOBP1 is reliable (Figure 7B–D). The predicted structure of SvelOBP1 has six *α*-helices (*α*1 located at residues from Pro6 to Ser23, *α*2 located at residues from Glu27 to Ala32, *α*3 located at residues from Gln40 to Glu52, *α*4 located at residues from Tyr64 to Gln70, *α*5 located at residues from Pro73 to Asn84, and *α*6 located at residues from Leu95 to Ala109). The three internal disulfide bridges (Cys19-Cys48, Cys44-Cys96, and Cys86-Cys105) stabilized the tertiary structure.

**Figure 8:**
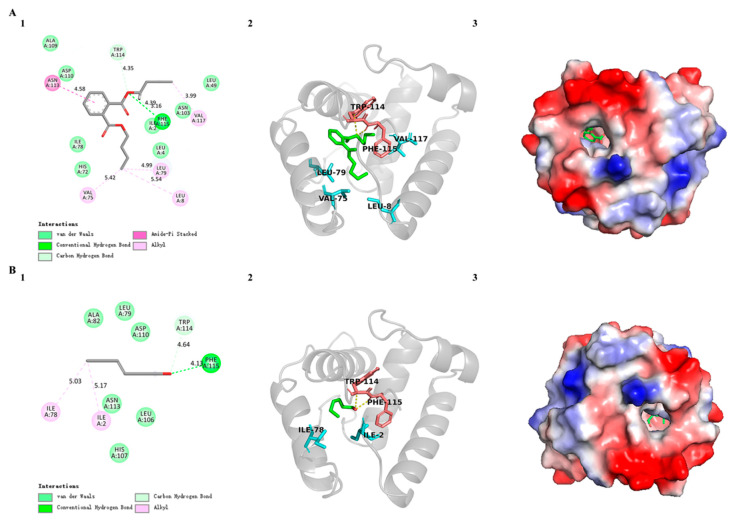
Docking results of SvelOBP1 with (**A**) dibutyl benzene-1,2-dicarboxylate and (**B**) (*Z*)-hex-3-en-1-ol. (1) Two-dimensional predicted interactions of SvelOBP1 with ligand. (2) Three-dimensional predicted interactions of SvelOBP1 with ligand. The ligand is marked in green, and the residues formed by hydrogen bonds are marked in red. (3) The electrostatic potential surface of SvelOBP1 binding with ligand. Red: negative; white: neutral; blue: positive.

In order to further investigate the binding sites of SvelOBP1 with odorants and to validate the result of the binding assay, two ligands with the strongest binding ability were selected (dibutyl benzene-1,2-dicarboxylate and (*Z*)-hex-3-en-1-ol) to dock with the SvelOBP1 protein. The docking results displayed two ligands bound with SvelOBP1 at negative energy values of −6.71 and −6.28 kcal/mol, respectively. In addition, dibutyl benzene-1,2-dicarboxylate and (*Z*)-hex-3-en-1-ol were located in the same pocket consisting of some hydrophobic residues. The key residues participated in the binding with SvelOBP1 and both ligands are visualized in Figure 8. Hydrogen bonds, hydrophobic interactions and Van Der Waals forces were vital in binding with SvelOBP1 and these ligands. Dibutyl benzene-1,2-dicarboxylate is one of the esters. Hydrogen bonds were formed by residues Trp114 and Phe115 in SvelOBP1 with the dibutyl benzene-1,2-dicarboxylate. Hydrophobic interactions (Leu8, Val75, Leu79, Asn113 and Val117) and Van Der Waals interactions (Ile2, Leu4, Leu49, His72, Ile78, Asn103, Ala109 and Asp110) were also involved in molecular docking between SvelOBP1 and dibutyl benzene-1,2-dicarboxylate. On the other hand, (*Z*)-hex-3-en-1-ol is one of the highly hydrophobic alcohols. Similar interactions were found between SvelOBP1 and (*Z*)-hex-3-en-1-ol. Trp114 and Phe115 in SvelOBP1 formed hydrogen bonds with (*Z*)-hex-3-en-1-ol. The residues Ile2 and Ile78 were involved in hydrophobic interactions. The residues Leu79, Ala82, Leu106, His107, Asp110 and Asn113 were involved in Van Der Waals forces.

Additionally, we performed molecular docking of SvelOBP1 with 24 ligands, and the key amino acid residues involved in different interactions are listed in Table S1. The results showed that K_i_ values ranged from −6.71 to −3.45 kcal/mol and were highly significantly correlated with the binding energies (r = 0.722, *p* < 0.01). Different ligands usually had different binding characteristics. Compared with other groups of chemical compounds, terpenoids, except 3,7,7-trimethylbicyclo[4.1.0]hept-3-ene, showed lower binding energies (<−5 kcal/mol) and there was no hydrogen bond found in the binding with SvelOBP1. Ala82, Leu106 and Trp114 were found to be involved in the hydrophobic interaction during the binding process of five terpenoids with SvelOBP1. Both Asp110 and Asn113 were involved in Van der Waals forces during the binding process of the five terpenoids with SvelOBP1 (Table S1).

## 4. Discussion

In this study, the *SvelOBP*1 gene of *S. velatus* was cloned and characterized. The predicted mature protein has a signal peptide with 18 amino acids at the N-terminal. SvelOBP1 displayed the typical characterization of insect OBPs, with six conserved cysteines. The secondary structure of SvelOBP1 contained six *α*-helices and three disulfide bridges. Sequence alignments and phylogenetic analyses indicated that SvelOBP1 was classified into the classic OBP subfamily and shared high sequence identity with other coleopteran insects. The closest homolog of SvelOBP1 was PyasOBP2 from *P. yasumatsui* (80.91% sequence identity). SvelOBP1 was one of the most abundant OBPs in the transcript level of *S. velatus*. The expression level of OBPs is interrelated to their physiological functions [31]. Based on the expression patterns, the dominant body part expressing *SvelOBP*1 was the antennae and the expression of *SvelOBP*1 was higher in male antennae than that in female antennae. On the other hand, the expression in other body parts of both sexes was negligible (head, thorax, abdomen, leg and wing). Previous studies have shown that OBPs are mainly expressed in the antennae and play a vital role in the detection and chemoreception of volatile chemicals [45]. Based on the results, we speculated that SvelOBP1 could play similar roles in the olfaction perception of *S. velatus*.

To confirm the potential roles of SvelOBP1 in the detection of the host plant volatiles, the competitive fluorescence binding assay of recombinant SvelOBP1 protein was evaluated. SvelOBP1 could bind with 24 compounds (K_i_ < 16 μM) out of 43 tested volatiles, which indicates that SvelOBP1 has a broad ligand binding affinity for the volatiles emitted from jujube leaves. SvelOBP1 has distinct binding abilities to different ligands. Dibutyl benzene-1,2-dicarboxylate and (*Z*)-hex-3-en-1-ol presented the strongest binding affinity for SvelOBP1 (K_i_ = 6.66 ± 0.03 and 7.98 ± 0.06 μM). These two ligands were both abundant plant volatiles with high binding affinities to insect OBPs [44,46,47,48,49,50]. In Li’s study [31], the binding affinity of SvelOBP15 with 33 plant volatiles was determined. The results showed that SvelOBP15 could bind with four plant volatiles, and it hardly bound with (Z)-hex-3-en-1-ol (K_i_ > 16 μM). In this study, SvelOBP1 displayed a wider binding spectrum than SvelOBP15, and it showed strong binding ability with (Z)-hex-3-en-1-ol. Therefore, it is believed that different OBPs in *S. velatus* have different binding affinities to the same compound. Our study merely identified the binding capability of SvelOBP1 with host volatiles. Indeed, some OBPs also have the functions of binding sex pheromones [15,51,52], while the sex pheromones of *S. velatus* are unknown. Therefore, the function of SvelOBP1 involved in detecting sex pheromones still needs further investigation.

Some key residues located in the hydrophobic internal cavity were considered to contribute to odorant binding interactions between OBPs and ligands [27,53]. Dibutyl benzene-1,2-dicarboxylate and (*Z*)-hex-3-en-1-ol were selected to explore this specific binding feature with SvelOBP1 in this study. Hydrogen bonds, hydrophobic interactions and Van Der Waals forces were found in the binding of SvelOBP1 with both ligands. The residues Trp114 and Phe115 formed hydrogen bonds both in binding with dibutyl benzene-1,2-dicarboxylate and (*Z*)-hex-3-en-1-ol. Similar to the binding results of SvelOBP1, the C-terminal residues Tyr122 and Phe123 of AgamOBP1, which occupied the same positions as Trp114 and Phe115 of SvelOBP1 in the sequence alignment, also formed hydrogen bonds with MOP (a mosquito oviposition pheromone, (*5R*,*6S*)-6-acetoxy-5-hexadecanolide) [54]. In addition, the residue Asp110 in SvelOBP1 was mainly involved in Van Der Waals interactions. *Apolygus lucorum* AlucOBP22 [6], *Holotrichia oblita* HoblOBP1 [55], *Diaphorina citri* DcitOBP7 [56], *Harmonia axyridis* HaxyOBP3 [57] and *Bactrocera minax* BminOBP3 [58] were also reported to have these interactions with ligands. Therefore, it is believed that Trp114, Phe115 and Asp110 could be the key amino acid residues which are responsible for specific ligand binding.

In conclusion, the gene *SvelOBP1* was cloned and analyzed in this study. *SvelOBP*1 showed an antenna-specific expression profile. The SvelOBP1 protein demonstrated obvious selective binding characteristics with jujube volatiles. The key residues involved in the interactions of SvelOBP1 with dibutyl benzene-1,2-dicarboxylate and (*Z*)-hex-3-en-1-ol were characterized. Overall, this study elucidated the potential function of SvelOBP1 in recognizing host odorants of *S. velatus*, and SvelOBP1 might be a useful molecular target for *S. velatus* control.

## Figures and Tables

**Table 1:** Primers used for cloning and gene expression analyses in *S. velatus*.

Primer Name	Primer Sequences (5′-3′)	Fragment Length (bp)
**For gene cloning**		
*SvelOBP*1-F	ATGTCTAAAATTATAGGTCTGTTGG	408
*SvelOBP*1-R	TTASACRAAGAACCARTTCAC	
**For qRT-PCR**		
*SvelOBP*1-qF	GCTCCAAGAATACGTCGATGA	168
*SvelOBP*1-qR	TCGATTTCACCACTGGGTTT	
*EF1α*-qF	AACCACCATACAGCGAGTCC	128
*EF1α*-qR	CATGTTGTCTCCATGCCATC	
*βactin*-qF	CTACGAAGGTTACGCTCTTCCC	123
*βactin*-qR	CTTTCAGCTGTGGTGGTGAAT	
**For prokaryotic expression**		
*SvelOBP*1-eF	CGGGATCCAAGATAACTTTACCTCCTGAGCTCC	374
*SvelOBP*1-eR	CCGGAATTCCGGTTASACRAAGAACCAGTTCACAGG	

Note: Restriction sites are underlined.

**Table 2:** Binding affinities of SvelOBP1 to host plant volatiles from leaves of jujube trees.

Ligands	CAS No.	IC_50_ (μM)	K_i_ (μM)
**Esters**			
Ethyl butyrate	105-54-4	14.19 ± 0.16	11.79 ± 0.13
Butyl acetate	123-86-4	13.16 ± 0.47	10.94 ± 0.39
Ethyl (*E*)-2-methylbut-2-enoate	5837-78-5	>16	−
Ethyl pentanoate	539-82-2	14.12 ± 0.31	11.73 ± 0.26
Ethyl 2-methylbutyrate	7452-79-1	11.28 ± 0.10	9.37 ± 0.08
Ethyl 3-methylbutanoate	108-64-5	>16	−
Methyl 2-hydroxybenzoate	119-36-8	>16	−
[(*E*)-hex-2-en-1-yl] acetate	2497-18-9	>16	−
[(*Z*)-hex-3-enyl] acetate	3681-71-8	14.45 ± 0.11	12.01 ± 0.09
[(*Z*)-hex-3-enyl] butanoate	16491-36-4	15.52 ± 0.90	12.90 ± 0.75
[(*Z*)-hex-3-enyl] 2-methylbutanoate	53398-85-9	13.42 ± 0.20	11.15 ± 0.16
[(*Z*)-hex-3-enyl] 3-methylbutanoate	35154-45-1	14.37 ± 0.16	11.94 ± 0.13
[(*E*)-hex-2-enyl] hexanoate	53398-86-0	14.51 ± 0.06	12.06 ± 0.05
Bis(2-methylpropyl) hexanedioate	141-04-8	>16	−
Dibutyl benzene-1,2-dicarboxylate	84-74-2	9.60 ± 0.09	6.66 ± 0.03
**Alcohols**			
(*E*)-hex-2-en-1-ol	928-95-0	15.44 ± 0.14	12.83 ± 0.12
(*Z*)-hex-3-en-1-ol	928-96-1	10.51 ± 0.11	7.98 ± 0.08
Phenylmethanol	100-51-6	11.31 ± 0.27	9.40 ± 0.23
Oct-1-en-3-ol	3391-86-4	15.18 ± 0.13	12.61 ± 0.11
3,7-dimethylocta-1,6-dien-3-ol	78-70-6	13.59 ± 0.16	11.29 ± 0.13
1,3,3-trimethyl-2-oxabicyclo [2.2.2]octane	470-82-6	13.81 ± 0.13	11.48 ± 0.11
(2*E*)-3,7-dimethylocta-2,6-dien-1-ol	106-24-1	>16	−
(2*E*,6*E*)-3,7,11-trimethyldodeca-2,6,10-trien-1-ol	4602-84-0	14.36 ± 0.17	11.93 ± 0.14
(6*E*)-3,7,11-trimethyldodeca-1,6,10-trien-3-ol	7212-44-4	>16	−
(*E*)-3,7,11,15-tetramethylhexadec-2-en-1-ol	7541-49-3	>16	−
**Terpenoids**			
3,7,7-trimethylbicyclo [4.1.0]hept-3-ene	13466-78-9	14.56 ± 0.07	12.10 ± 0.06
2,2-dimethyl-3-methylidenebicyclo [2.2.1]heptane	79-92-5	>16	−
(1*S*,5*S*)-2,6,6-trimethylbicyclo [3.1.1]hept-2-ene	7785-26-4	>16	−
7-methyl-3-methylideneocta-1,6-diene	123-35-3	>16	−
3,7-dimethylocta-1,3,6-triene	13877-91-3	11.13 ± 0.06	9.25 ± 0.05
2-methyl-5-propan-2-ylcyclohexa-1,3-diene	99-83-2	11.69 ± 0.07	9.71 ± 0.06
1-methyl-4-prop-1-en-2-ylcyclohexene	7705-14-8	13.11 ± 0.02	10.90 ± 0.01
(3*E*,6*E*)-3,7,11-trimethyldodeca-1,3,6,10-tetraene	502-61-4	8.01 ± 0.04	8.73 ± 0.09
(6*E*,10*E*,14*E*,18*E*)-2,6,10,15,19,23-hexamethyltetracosa-2,6,10,14,18,22-hexaene	111-02-4	>16	−
**Others**			
1-(4-ethylphenyl)ethanone	937-30-4	13.13 ± 0.16	10.91 ± 0.13
1-(3,4-dimethylphenyl)ethanone	3637-01-2	>16	−
(2*E*)-3,7-dimethylocta-2,6-dienoic acid	459-80-3	>16	−
Hexadecanoic acid	57-10-3	14.09 ± 0.33	11.71 ± 0.27
2-cyclopentylcyclopentan-1-one	4884-24-6	>16	−
2-methylpropanal	78-84-2	>16	−
2-methoxy-4-prop-2-enylphenol	97-53-0	>16	−
Dodecane	112-40-3	>16	−
2-methylprop-1-enylbenzene	768-49-0	>16	−

The data are shown as “mean ± standard error of mean (SEM)”. “>16” indicates that the IC50 values were above the concentration ranges tested. “−” means that the Ki values were not calculated.

## Data Availability

Data are included in the main text and Supplementary Materials.

## Supplementary Materials

The following supporting information can be downloaded at: https://www.mdpi.com/article/10.3390/life14020192/s1, Figure S1: Nucleotide and deduced amino acid sequences of SvelOBP1 in *Sympiezomias velatus*. The signal peptides are underlined in red. The conserved cysteine residues are indicated in red boxes. Figure S2: Verifying the result of 3D model of SvelOBP1. Figure S3: The Ramanchandran plot of the 3D model of SvelOBP1. Table S1: Molecular docking of SvelOBP1 with 24 ligands.
