# Supplementary material for: The Sequence Characteristics and Binding Properties of the Odorant-Binding Protein SvelOBP1 from Sympiezomias velatus (Coleoptera: Curculionidae) to Jujube Volatiles
Source: Life (Basel). 2024 Jan 29;14(2):192. doi: 10.3390/life14020192 (PMC10890569; doi:10.3390/life14020192)
Supplement: Supplementary file 1 [file life-14-00192-s001.zip › Figure S1 Nucleotide and deduced amino acid sequences of SvelOBP1 in Sympiezomia velatus. .pdf]

```
1    ATGTCTAAAATTATAGGTCTGTTGGTATTAGTGTTGTTTACTGTTGGTAATGCTAAGATAACTTTACCTCCTGAGCTCCAAGAATACGTCGATGACTTACACAAACTATGTATTTCAAAA
1     M  S  K  I  I  G  L  L  V  L  V  L  F  T  V  G  N  A  K  I  T  L  P  P  E  L  Q  E  Y  V  D  D  L  H  K  L  C  I  S  K
121  AGTGGAATTACTGAGGATGACCACCAAGCTTATGATATATCCAATAAGGACCAAAAAATGATGTGCTATATGAAATGTCTTATGCTGGAGTCTAAGTGGATGAAACCCAGTGGTGAAATC
41    S  G  I  T  E  D  D  H  Q  A  Y  D  I  S  N  K  D  Q  K  M  M  C  Y  M  K  C  L  M  L  E  S  K  W  M  K  P  S  G  E  I
241  GATTATGCTTATATTGAAGAACAAGCTCATCCAGACGTAAAAGATATTTTAATGGCTGCTCTTAACACATGCAAAGTTATTAACGACGGAGCTGATCTTTGTGAAAAATCCTATAACTTC
81    D  Y  A  Y  I  E  E  Q  A  H  P  D  V  K  D  I  L  M  A  A  L  N  T  C  K  V  I  N  D  G  A  D  L  C  E  K  S  Y  N  F
361  AACCTCTGTCTTCATGAAGCTGACCCTGTGAACTGGTTCTTTGTGTAA
121   N  L  C  L  H  E  A  D  P  V  N  W  F  F  V  *
```
