# Supplementary material for: The Sequence Characteristics and Binding Properties of the Odorant-Binding Protein SvelOBP1 from Sympiezomias velatus (Coleoptera: Curculionidae) to Jujube Volatiles
Source: Life (Basel). 2024 Jan 29;14(2):192. doi: 10.3390/life14020192 (PMC10890569; doi:10.3390/life14020192)
Supplement: Supplementary file 1 [file life-14-00192-s001.zip › Figure S3 The Ramanchandran plot of the 3D model of SvelOBP1.pdf]

PROCHECK

Ramachandran Plot

saves

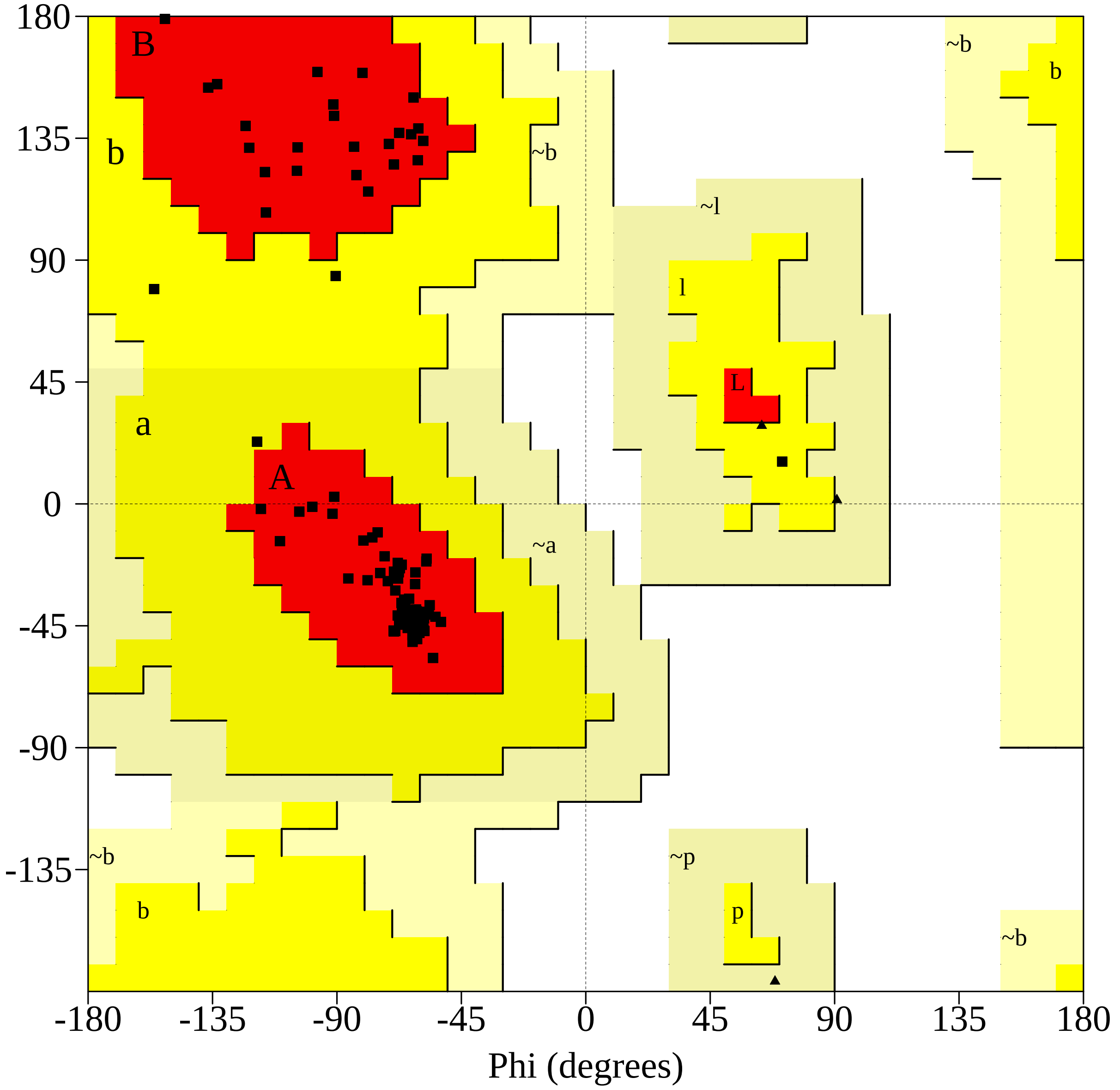

Plot statistics

Residues in most favoured regions [A,B,L]	103	96.3%
Residues in additional allowed regions [a,b,l,p]	4	3.7%
Residues in generously allowed regions [~a,~b,~l,~p]	0	0.0%
Residues in disallowed regions	0	0.0%
	----	------
Number of non-glycine and non-proline residues	107	100.0%
Number of end-residues (excl. Gly and Pro)	2	
Number of glycine residues (shown as triangles)	3	
Number of proline residues	5	

Total number of residues	117	

Based on an analysis of 118 structures of resolution of at least 2.0 Angstroms and R-factor no greater than 20%, a good quality model would be expected to have over 90% in the most favoured regions.

saves_01.ps
