# Supplementary material for: The Sequence Characteristics and Binding Properties of the Odorant-Binding Protein SvelOBP1 from Sympiezomias velatus (Coleoptera: Curculionidae) to Jujube Volatiles
Source: Life (Basel). 2024 Jan 29;14(2):192. doi: 10.3390/life14020192 (PMC10890569; doi:10.3390/life14020192)
Supplement: Supplementary file 1 [file life-14-00192-s001.zip › TableS1 Molecular docking of SvelOBP1 with 24 ligands.pdf]

Supplementary Materials

Table S1 Molecular docking of SvelOBP1 with 24 ligands

Number	Ligands	K_i(μM)	Binding energy (kcal/mol)	Hydrogen bond	Hydrophobic interaction	Van der Waals force
**Esters**						
1	Dibutyl benzene-1,2-dicarboxylate	6.66 ± 0.03	-6.71	Trp114,Phe115	Leu8,Val75,Leu79,Asn113,Val117	Ile2,Leu4,Leu49,His72,Ile78,Asn103,Ala109,Asp110
2	Ethyl 2-methylbutyrate	9.37 ± 0.08	-5.98	–	Leu15,Leu49,Met50,Trp55,Phe102,Leu106	Tyr11,Val12,Met46,Ser53,Asn103,Phe116,Val117
3	Butyl acetate	10.94 ± 0.39	-3.68	Ile67	Leu8,Ala71,His72,Val75,Leu79	Tyr11,Ser53,Trp55,Tyr66,Gln70
4	[(*Z*)-hex-3-enyl] 2-methylbutanoate	11.15 ± 0.16	-5.28	Asp110,Leu106	Ile2,Val75,Ile78,Leu79,Trp114,Phe115	Trp55,Ala82,Asn103,Ala109,Val117
5	Ethyl pentanoate	11.73 ± 0.26	-3.97	Trp114	Ala82,Leu106,Phe115	Ile2,Ile78,Leu79,His107,Ala109,Asp110,Asn113
6	Ethyl butyrate	11.79 ± 0.13	-3.67	–	Val2,Tyr11,Leu15,Met50,Trp55,Phe102,Val17	Ser53,Mey46,Asn103,Leu49,Leu106,
7	[(*Z*)-hex-3-enyl] 3-methylbutanoate	11.94 ± 0.13	-4.85	Trp114,Phe115	Leu49,Met50,Ala82,Leu106	Ile2,Met46,Trp55,Leu79,Ile78,Asn103,His107,Asp110,Asn113,Val117,Phe116

8	[(*Z*)-hex-3-enyl] acetate	12.01 ± 0.09	-4.43	–	Phe115,Val117	Ile2,Trp55,Ile78,Leu79,Ala82,Leu106,Asp110,Asn113,Trp114,Phe116
9	[(*E*)-hex-2-enyl] hexanoate	12.06 ± 0.05	-5.38	His72	Ile2,Trp55,Tyr66	Leu4,Leu8,Ser53,Ile67,Gln70,Ala71,Val75,Ile78,Leu79,Ala82,Asp110,Asn113,Trp114,Phe115
10	[(*Z*)-hex-3-enyl] butanoate	12.90 ± 0.75	-4.48	Leu79,Asp110	Ile2,Val75,Trp114,Phe115	Leu4,Leu8,Trp55,Ile78,Ala82,Leu106,Ala109,Asn113
**Alcohols**						
11	(*Z*)-hex-3-en-1-ol	7.98 ± 0.08	-6.28	Trp114,Phe115	Ile2,Ile78	Leu79,Ala82,Leu106,His107,Asp110,Asn113
12	Phenylmethanol	9.40 ± 0.23	-4.10	Phe115	Ala82,Asn113	Ile2,Ile78,Leu79,Leu106,Ala109,Asp110,Trp114
13	3,7-dimethylocta-1,6-dien-3-ol	11.29 ± 0.13	-5.12	Asp110	Ala82,Leu106,Trp114,Phe115	Ile2,Leu4,Trp55,Ile78,Leu79,Leu83,His107,Asn113,Val117
14	1,3,3-trimethyl-2-oxabicyclo[2.2.2]octane	11.48 ± 0.11	-5.48	–	Ile2,Leu79,Ala82,Leu106,Trp114,Phe116	Ile78,Leu83,Ala109,Asp110,Asn113
15	(2*E*,6*E*)-3,7,11-trimethyldodeca-2,6,10-trien-1-ol	11.93 ± 0.14	-4.09	Tyr66,Ile67	Tyr11,Val12,Leu49,Mey50,Trp55,Ala71,Leu79,Val117	Leu8,Leu15,Met46,Ser53,Gln70,His72,Asn103,Leu106
16	Oct-1-en-3-ol	12.61 ± 0.11	-4.39	Leu106	Ile2,Ile78,Phe115,Val117	Trp55,Leu79,Ala82,Leu106,His107,Ala109,Asp110,Asn113,Trp114
17	(*E*)-hex-2-en-1-ol	12.83 ± 0.12	-3.61	Phe115	Ile2,Ile78	Leu79,Ala82,Leu106,Asp110,Asn113,Trp114
**Terpenoids**						
18	(3*E*,6*E*)-3,7,11-trimethyldodeca-1,3,6,10-tetraene	8.73 ± 0.09	-6.41	–	Ile2,Leu15,Leu49,Met50,Trp55,Ile78,Ala82,Leu106,Trp114,Phe115,Val117	Met46,Ser53,Leu79,Phe102,Asn103,His107,Asp110,Asn113,Phe116

19	3,7-dimethylocta-1,3,6-triene	9.25 ± 0.05	-5.27	–	Trp55,Ile78,Leu79,Ala82,Leu106,Trp114,Val117	Ile2,Asn103,His107,Ala109,Asp110,Asn113,Phe115,Phe116
20	2-methyl-5-propan-2-ylcyclohexa-1,3-diene	9.71 ± 0.06	-5.36	–	Ile78,Ala82,Leu106,Trp114	Ile2,Leu79,Asn103,Asp110,Asn113,Phe115,Phe116,Val117
21	1-methyl-4-prop-1-en-2-ylcyclohexene	10.90 ± 0.01	-5.21	–	Ala82,Leu106,Trp114,Val17	Ile2,Trp55,Ile78,Leu79,Asn103,His107,Asp110,Asn113,Phe115,Phe116
22	3,7,7-trimethylbicyclo[4.1.0]hept-3-ene	12.10 ± 0.06	-3.46	–	Ile2,Ile78,Ala82,Leu106,Trp114,Phe115	Trp55,Leu79,His107,Ala109,Asp110,Asn113
**Others**						
23	1-(4-ethylphenyl)ethanone	10.91 ± 0.13	-5.48	Trp55	Leu8,Gln70,Ala71,Val75,Leu79	Leu4,Tyr11,Ser53,Tyr66,Ile67,His72
24	Hexadecanoic acid	11.71 ± 0.27	-3.45	–	Ala82,Leu106,Trp114	Ile2,Leu8,Trp55,Ser53,Tyr66,Ile67,Gln70,Ala71,His72,Val75,Ile78,Leu79,His107,Ala109,Asp110,Asn113
