# Supplementary figures and images for: The Sequence Characteristics and Binding Properties of the Odorant-Binding Protein SvelOBP1 from Sympiezomias velatus (Coleoptera: Curculionidae) to Jujube Volatiles
Source: Life (Basel). 2024 Jan 29;14(2):192. doi: 10.3390/life14020192 (PMC10890569; doi:10.3390/life14020192)

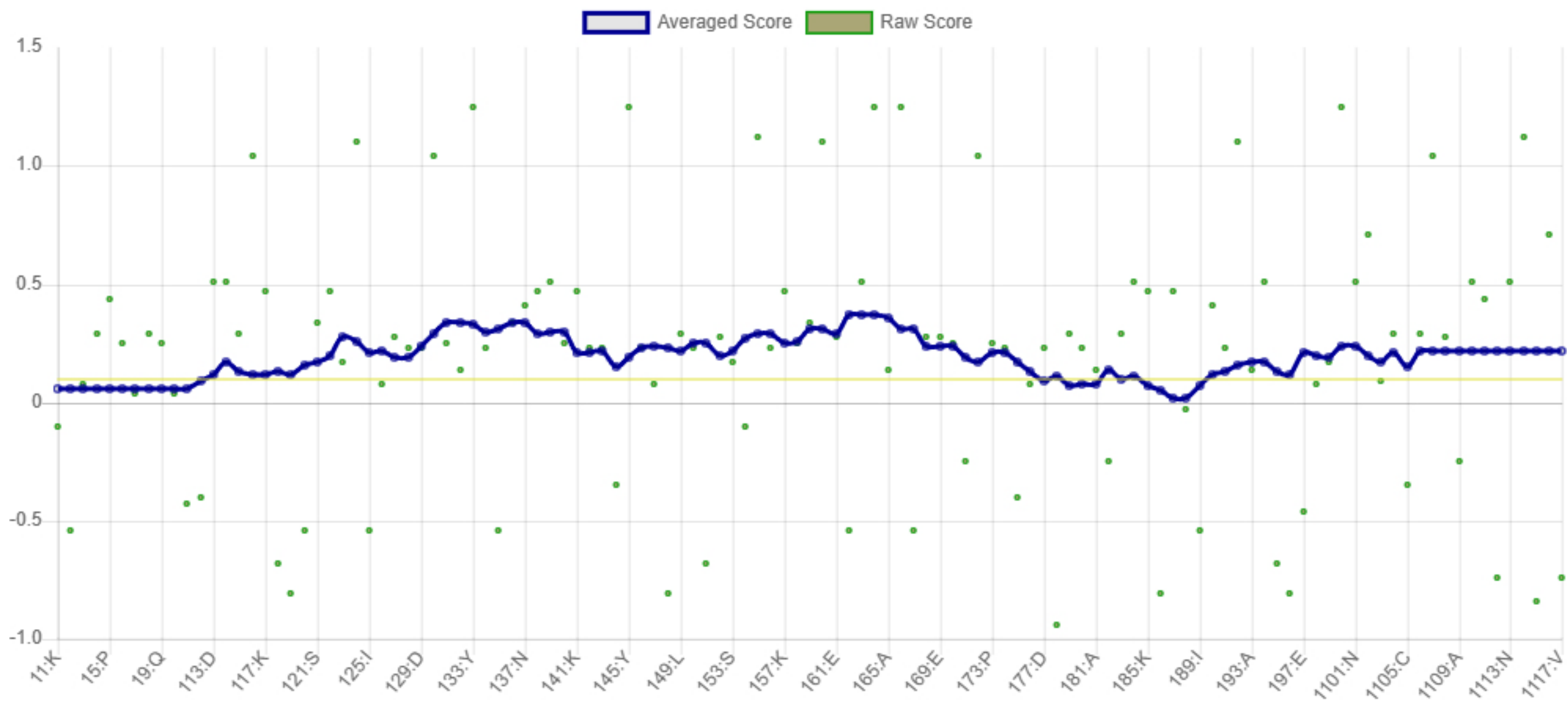

Supplement: Supplementary file 1 [file life-14-00192-s001.zip › Figure S2 Verifing the result of 3D model of SvelOBP1.pdf]
